# Co-Design of an Evidenced Informed Service Model of Integrated Palliative Care for Persons Living with Severe Mental Illness: A Qualitative Exploratory Study

**DOI:** 10.3390/healthcare9121710

**Published:** 2021-12-09

**Authors:** Marianne Tinkler, Joanne Reid, Kevin Brazil

**Affiliations:** 1 Northern Health and Social Care Trust, Antrim BT41 2RL, UK; mtinkler01@qub.ac.uk; 2 Medical Biology Centre, School of Nursing and Midwifery, Queen’s University Belfast, Belfast BT9 7BL, UK; k.brazil@qub.ac.uk

**Keywords:** qualitative research, co-design, severe mental illness, palliative care, end-of-life care

## Abstract

Background: Globally, close to one billion people are living with a mental health disorder, and it is one of the most neglected areas in Public Health. People with severe mental illness have greater mortality risk than the general population, experience health care inequalities throughout life and represent a vulnerable, under-served and under-treated population, who have been overlooked in health inequality research to date. There is currently a dearth of evidence in relation to understanding the palliative care needs of people with severe mental illness and how future care delivery can be designed to both recognise and respond to those needs. This study aims to co-design an evidenced informed service model of integrated palliative care for persons living with a severe mental illness. Methods: This qualitative sequential study underpinned by interpretivism will have six phases. An expert reference group will be established in Phase 1, to inform all stages of this study. Phase 2 will include a systematic literature review to synthesise current evidence in relation to palliative care service provision for people with severe mental illness. In Phase 3, qualitative interviews will be undertaken with both, patients who have a severe mental illness and in receipt of palliative care (n = 13), and bereaved caregivers of people who have died 6–18 months previously with a diagnosis of severe mental illness (n = 13), across two recruitment sties in the United Kingdom. Focus groups (n = 4) with both mental health and palliative care multidisciplinary staff will be undertaken across the two recruitment sites in Phase 4. Phase 5 will involve the co-design of a service model of integrated palliative care for persons living with severe mental illness. Phase 6 will develop practice recommendations for this client cohort. Discussion: Palliative care needs to be available at all levels of care systems; it is estimated that, globally, only 14% of patients who need palliative care receive it. Reducing inequalities experienced by people with severe mental illness is embedded in the National Health Service Long Term Plan. Internationally, the gap between those with a mental illness needing care and those with access to care remains considerable. Future policy and practice will benefit from a better understanding of the needs of this client cohort and the development of a co-designed integrated care pathway to facilitate timely access to palliative care for people with a severe mental illness.

## 1. Background

People with a severe mental illness (SMI) face significant inequalities with regard to their physical and mental health, often facing stigma and exclusion. The World Health Organisation (WHO) defines mental illness as depression, bipolar disorder, schizophrenia and other psychoses [1]. This population are often diagnosed with terminal conditions much later than members of the general population [2]. Severe mental illness (SMI) is any psychiatric disorder that is not transient (i.e., it persists for a long period) that requires care/treatment and is associated with severe social and societal limitations [3]. Previous research has highlighted the palliative care needs of people with SMI are under-recognised and under-treated [4,5], occurring most frequently only in relation to crisis management. Thus, understanding the palliative care needs of people with SMI and developing services which recognise and respond to their needs is required [3].

While people with SMI have a high need for palliative care [6], they face inequalities in healthcare and are seen as a vulnerable population, underrepresented in health disparities research [7]. This is reflective of a “loud silence”, and neglect, when it comes to SMI and death and dying [8].

Adults with SMI experience higher rates of physical ill health and have a life expectancy 10–20 years lower than the general population [9]. Excess mortality is primarily due to chronic diseases such as cancer, heart disease, chronic obstructive pulmonary disease, and dementia [10,11]. Excess mortality from chronic disease is multifactorial; people with schizophrenia, for example, have similar incidence of most cancers to the general population, but have up to a two-fold risk of dying of their disease due to under-detection and under-treatment [8].

The benefits of palliative care span multiple domains including improvements in symptom burden and quality of life for people and caregivers [12]. Such benefits are most notable with early provision of palliative care. Evidence suggests early palliative care, often in conjunction with disease-oriented treatment, can profoundly affect the trajectory of illness by improving quality of life and mood and decreasing aggressive end-of-life interventions [13].

In the United Kingdom, policy on palliative and end-of-life care embeds the idea that as part of a humane society, dying well no matter where or who you are, whatever the circumstances, is a basic aspect of human dignity [14]. In line with the end-of-life care strategy, the Department of Health’s Service Framework for Mental Health and Wellbeing [15] identified palliative care as a generic standard. Despite the high chronic disease morbidity and mortality experienced by people with an SMI, data suggest that the provision of palliative care to these people is poor. Lack of access to palliative care directly affects the management of end-of-life symptoms. For example, patients with schizophrenia receive significantly less opioid analgesia and experience poor quality end-of-life care compared to patients without an SMI [4]. Palliative care disparities among people with an SMI are not limited to direct end-of-life care; a study comparing mentally ill and non-mentally ill nursing home residents found mental illness was associated with a 24% odds reduction of a resident having any form of advance care planning [16].

For this project, integrated palliative care is defined as bringing together administrative, organisational, clinical and service aspects in order to realise continuity of care between all actors involved in the care networks of people receiving palliative care [17]. It aims to achieve quality of life and supportive palliative care for the patient and the persons closest to them in collaboration with all caregivers. However, despite the popularity of developing integrated models of care, there is poor shared language across palliative care and mental health care disciplines [18].

It is acknowledged that palliative care for people with SMI poses unique challenges. There can be stigma and misattribution of physical symptoms due to having a mental illness; diagnostic overshadowing is a form of systemic disparity with negative language and marginalisation around people with SMI [19]. In addition, a lack of integration of services, all pose a threat to access to palliative care. A recent scoping review of palliative care and SMI confirmed that there cannot be an expectation of leaders and healthcare providers to deliver high quality integrated care without the voice of service users [20]. People with lived experience of SMI and their caregivers have a powerful and essential voice to provide an insight into the barriers and challenges experienced in accessing palliative care. To date, there has not been a development of an integrated service model of palliative care which responds to the needs of this under-serviced and under-researched group. Access to palliative care is emerging as a public health priority with a global need for palliative care to double over the next four decades [21]. Thus, the generation of recommendations from this study will inform policy and further research and service development within this area.

## 2. Methods

### 2.1. Aim

This study aims to co-design an evidenced informed service model of integrated palliative care for people living with an SMI.

Operational definition of severe mental illness (SMI)

Within this study, there is recognition of the lack of an accepted consensus definition for SMI. However, it was necessary to have an operational definition, and within this study, people with SMI are defined as individuals who experience reduced general functioning due to their psychiatric disease, being in need of specialist care and having received such care for at least two years [3,22,23,24]. Hypothetically, all psychiatric diagnoses could present with SMI; however, the major diagnosis groups are schizophrenia, bipolar disorder and personality disorder [24].

### 2.2. Study Design

This is an exploratory qualitative sequential study [25], based on interpretivism [26]. An exploratory (i.e., emergent theory) approach is one that requires the researcher to inductively and empirically develop theory from research data. The philosophy of interpretivist research is to understand the world from the point of view of the participants, rather than the world’s explanation; it is, therefore, consistent with the aim of the study.

Commonly, the PICo framework, (population, interest and context) can aid and guide the literature review maintaining relevancy with the research question at hand, translating these elements of the research question is the core of evidence in this instance. Due to the qualitative nature of this programme of work, the PEO framework (P—Population, people with SMI, E—Exposure, palliative care and O—Outcome, integrated care) is utilised, the appropriateness of which is outlined in the literature [27], to allow for a better understanding of the phenomena and experiences.

**Figure 1:**
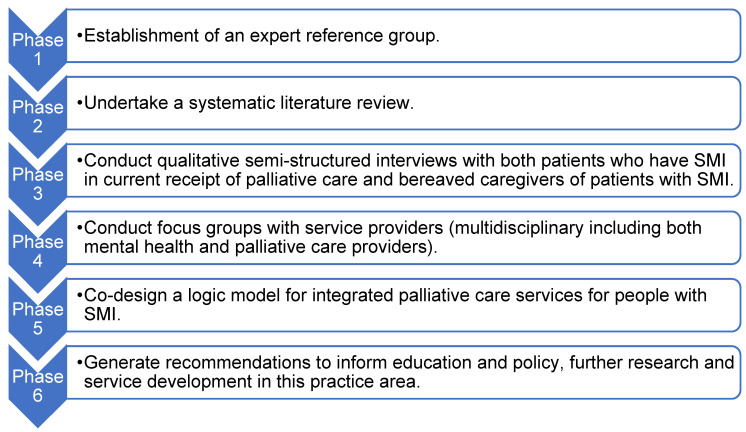
Overview of the six stages of the study.

There are six stages within this study, as outlined in Figure 1. The timeline of duration of each phase is as follows: establishment of expert reference group, 0–4 months with meetings at 12/24/30 months. Literature review, 0–9 months. Semi-structured interviews, 13–19 months. Development of a service model, 25–28 months. Recommendations from the study and expert reference group, 27–29 months.

Phase 1: an expert reference group will be established and consulted at all stages of this research project, in keeping with the co-design methodology incorporated into this study. The expert reference groups will comprise core stakeholder groups, including people with SMI and bereaved family caregivers, representatives of advocacy groups, health and social care professionals, policy makers, researchers and clinicians in palliative care and international experts in the field of study. The expert reference group will advise on the iterative stages within the research process, such as the development of interview (Phase 3) and focus groups (Phase 4) schedules. They will also review the qualitative study findings (Phases 3–5) and translate findings into the development of a co-designed service model for integrated palliative care services for people with SMI (Phases 5). Finally, they will help advise on proposed study recommendations (Phase 6) and guide a range of dissemination activities resulting from this study.

Phase 2: this will involve undertaking a systematic literature review [28]. The purpose of the systematic literature review will be to synthesise research conducted into palliative care and SMI to identify current evidence related to the palliative care needs for people with SMI, current service provision for this client cohort and evidence gaps in current care provision of this patient cohort. The review also contributes to the interview and focus group guides used in Phases 3 and 4.

Phase 3: this includes qualitative semi-structured interviews with both people who have SMI in current receipt of palliative care and bereaved caregivers of people with SMI. As previously outlined, many people with SMI do not access palliative care, and for those that do, who may be at the end stage of their lives, taking part in research may be difficult due to the increased deterioration and symptom burden. Recruiting only people with SMI as participants in palliative care research has the potential to be biased, as the patient may only be able to take part in the research if they are relatively well [29]. The challenges faced by people who are increasingly debilitated due to the impact of their disease may not be fully captured within the data. This is a complex recruitment challenge and the research team will adopt a two-pronged approach to data collection, recruiting both people with SMI currently in receipt of palliative care and bereaved caregivers of people with SMI. Bereaved caregivers have been chosen to be involved in this study as proxies for people with SMI in receipt of palliative care to provide vital insight into the person with SMI end-of-life care. Evidence shows that such proxies can reliably provide information on the person’s experience and quality of palliative care services and on patient’s symptoms at the end of their life [30,31]. By using this combined recruitment strategy, it is envisaged that the research team will gain a full understanding of the totality of the experience of palliative care provision for people with SMI. Caregiver participants will be recruited 6–18 months post-bereavement. The period of time post bereavement has been given careful consideration, and although this was not straight after the death of the person with SMI, it is within a time-frame which previous research has demonstrated allows bereaved caregivers to remember their experience, whilst minimising trauma to the participant [30] and giving due consideration to the ethical concerns when approaching bereaved caregivers [32]. Inclusion and exclusion criteria are detailed in Table 1 below.

We will conduct approximately 13 interviews with each group, i.e., people who have an SMI and are currently in receipt of palliative care and bereaved caregivers of people who have had an SMI. As this is an exploratory qualitative study, the final sample size will be determined by data saturation, the appropriateness of which is outlined in the literature [33]. The principle of 10 + 3 for data saturation outlines a minimum of 10 interviews should be conducted, followed by at least three consecutive interviews that present no new findings [34]. Thus, sample size will be determined by ‘information redundancy’ [35]. Recruitment will be from two Health and Social Care Trusts within the United Kingdom.

People with SMI currently in receipt of palliative care will be invited to take part in this research via a clinical gatekeeper. Gatekeepers are responsible for ensuring ethical standards are adhered to when selecting potential participants [36]. In this instance, the gatekeepers will be psychiatrists and community psychiatric nurses in each Health and Social Care Trust. Contact will be via outpatient clinics and domiciliary visits, while gatekeepers will provide (1) an invitation letter to take part in the study, (2) a participant information sheet and (3) a consent to be contacted form, which will be returned to the research team via a pre-paid envelope.

Bereaved caregivers will be invited to take part in this research via the lead community psychiatric nurses and a mental health caregivers advocacy group in each recruitment site. These individuals will act as gatekeepers to identify potential participants and contact them to invite them to take part in this study. Contact with potential bereaved caregiver participants will be via post; gatekeepers will provide (1) an invitation letter to take part in the study, (2) a participant information sheet and (3) a consent to be contacted form, which will be returned to the research team via a pre-paid envelope.

Once a consent to be contacted form has been received from either a patient or bereaved caregiver, the research team will arrange an interview on a date and time suitable for the participant. Each semi-structured interview will be conducted with participants in a location of their choosing to avoid the power dynamic present in a clinical/academic setting [37]. Considering the ongoing COVID-19 pandemic, face-to-face or telephone interviews lasting approximately one hour will be undertaken. Previous palliative care research has confirmed that telephone interviews do not lose depth of data [30,38]. An interview guide commensurate with guidelines for semi-structured interviews [39] will be utilised for each set of participants, informed by the literature and refined by the expert reference group. The interview guides will ensure participants can express and explore perspectives they may consider relevant to this topic area. The importance of using the right kind of interview questions to generate data has been highlighted in the literature. In acknowledging this, non-directive open-ended questions will be used to encourage and facilitate communication [40]. Icebreaker questions will be used to ease the participants into the semi-structured interviews, followed by the main topic questions relating to the study, concluding with closing questions to provide participants with the opportunity to raise any issues or air any concerns [41]. Questions will be based on the experience, feelings and knowledge of the participants [42], e.g., could you tell me about…? What understanding do you have of...? Probe questions will also be used throughout the semi-structured interview/focus groups as appropriate e.g., ‘can you tell me a little more about…?’ considering this good practice to avoid the researcher losing vital information [43]. All interviews will be digitally recorded and transcribed verbatim for analysis. We are aware the interview process will have the potential to cause distress; this has been addressed within the ethical considerations section of this protocol.

Phase 4: this will consist of focus groups with service providers; the participants for Phase 4 will be selected as healthcare professionals involved in the care of people with SMI and/or palliative care in the two recruitment sites. Six to eight multidisciplinary participants will be recruited to take part in each focus group, allowing for generating new insights and idea [44]. There will be at least two focus groups at each site, allowing for substantial discoverable themes [45]. The eligibility criteria for Phase 4 are outlined in Table 2.

The aim of the focus groups is to promote synergy by encouraging participants to comment, explain, disagree and share their views on their experiences of people with SMI and palliative care. This enables experiences to be shared that would not be possible in individual interviews, allowing for multidisciplinary communication from all who care for this cohort of individuals [46]. Clinical gatekeepers who are the heads of department for mental health and palliative care directorates within each recruitment site will be used to identify and make initial contact with potential participants. Contact will be via email or post, and they will provide (1) an invitation letter to take part in the study, (2) a participant information sheet and (3) a consent to be contacted form, which will be returned to the research team via a pre-paid envelope or via email.

Once consent to be contacted forms have been received, the research team will arrange focus groups on dates and times suitable for the participants. The focus groups will take place in a location convenient for all participants. In light of the ongoing COVID-19 pandemic, this may be via an online platform such as MS Teams, Zoom, etc. The appropriateness of using an online medium and confirmation that depth of data is maintained within online focus groups is provided from the literature [47]. Each focus group will last approximately one hour. Different members of the multidisciplinary teams, such as palliative care nurses, mental health nurses, palliative care doctors and consultants, psychiatrists and psychologists from each specialism (palliative care and mental health), will be recruited into the focus groups in order to provide a range of different perspectives. A focus group schedule will be used [48], which will be informed by the findings from Phases 2 and 3 and refined by the expert reference group. As discussed in relation to the semi-structured interviews in Phase 3, the research team will be cognisant of using non-directive open-ended questions to generate data. All focus groups will be digitally recorded and transcribed verbatim for analysis.

Phase 5: this will be the co-designing of a service model for integrated palliative care services for people with SMI. The best way to ensure an intervention effectively meets the needs of a target population is to involve key stakeholders in its design and development—this process is known as co-design [49]. A co-design approach in this project emphasises a partnership between the research team, caregivers of people with SMI, professionals and policy makers. The rationale to co-design a service model as a blueprint for the development of a future service model, rather than simple narrative recommendations alone, evolved from the literature [50] and fits with the aim of this study. A service model design incorporates functionality (how well it performs or is fit for purpose), safety (how safe and reliable it is) and usability (how interaction with the service or product is experienced) [50]. The service model employed in this study will be based on the Wisconsin Model, which is one of the most commonly used configurations for service modelling [51]. To co-design the service model for integrated palliative care services for people with SMI, the research team will conduct three workshops with the expert reference group. All workshop discussions will be digitally recorded and transcribed verbatim for analysis. In workshop one, an overview of the findings from phases 2–4 will be represented to workshop attendees to aid the development of an initial co-designed service model for integrated palliative care services for people with SMI. Data from this workshop will inform workshop 2, which will present the initial co-designed service model and seek refinement of this from the stakeholders. Data from workshop 2 will inform workshop 3, which will present a final co-designed service model for integrated palliative care services for people with SMI and seek agreement on this model from the stakeholders. The final service model will offer a visual, diagrammatic representation of what palliative needs are present for the person living with an SMI, what support they should receive and what it should look like, using the evidence generated from this study. The service model will identify the service components, processes, activities and desired outputs to improve the quality of care for this target population, including inputs (funding, training and staff time), outputs (activities and inter-/intra-organisational relations) and short- to long-term outcomes, whilst taking into consideration external influences and assumptions [51]. It is anticipated the workshops and all expert reference group meetings will run face to face; however, the research team is again mindful of COVID-19 and will be flexible and inclusive. If required, attendees can join meetings via an online platform (such as MS Teams or Zoom).

Phase 6: this will be the generation of recommendations to inform education and policy, further research and service development in this practice area. The findings of research will positively contribute to practice, knowledge generation and patient care and draw out the implications of this research for practitioners, educators, policymakers and researchers. In this phase, the research team will ensure systematic identification of recommendations relevant to understanding the needs of this population from the perspective of people with SMI, caregivers, health care providers and policy makers. They will also outline future research recommendations in relation to implementing the co-designed service model for integrated palliative care services for people with SMI. The RE-AIM (reach, effectiveness, adoption, implementation and maintenance) framework [52] will guide an iterative line of inquiry between the research team and the expert reference group. The RE-AIM framework was originally developed to support the planning and evaluation of health care interventions, and the appropriateness of using this framework within the development of a complex healthcare intervention is outlined in the literature [53]. The five constructs of the framework are considered important for effective and sustainable implementation of service interventions. In this phase of the project, the RE-AIM framework will enable a systematic identification of recommendations relevant to implementing the developed service model.

### 2.3. Analysis (Phases 3–5)

Qualitative data from people with SMI and bereaved caregivers’ interviews (Phase 3), multi-disciplinary focus groups (Phase 4) and co-design workshops (Phase 5) will be analysed using thematic analysis, with each phase of analysis conducted independently. This inductive approach to analysis will follow the six stages outlined [54]. Step 1 involves familiarisation with the data; it will start with listening to the digital recordings and reading the transcripts for accuracy, followed by reading and re-reading the transcripts to become familiar with the data. Stage 2 will generate initial codes; these will be developed through assigning a phrase or concept to a portion of the data to define its meaning in relation to the data generated and the research aim [55]. Stage 3 will begin when all data have been initially coded and collated and will search for themes within the data by considering how different codes combine together to form themes. Stage 4 will review themes and refine them to ensure they clearly explain the data collected. Stage 5 is to clearly define what each theme is, and what it is not, by developing names (descriptive labels) for each theme. Finally, stage 6 will begin when the research team has a set of fully worked-out themes. This stage involves telling the complicated story of the data in a way which convinces a reader of the merit and validity of the analysis. The importance of providing a coherent, nonrepetitive account of the data and thoughtfully selecting quotations to represent the findings is recognised [54]. In each stage of data analysis, the lead researcher (MT) will work closely with team members (J.R. and K.B.) who will read a proportion (30%) of the transcripts and independently code them, the appropriateness of which is outlined in the literature [56]. NVIVO will be used for Phases 3–5 data management [57]. All data display tables and any visual representations of the evolving codes and themes within NVIVO will be retained for the audit trail of this analysis.

### 2.4. Rigour

The criteria proposed by Lincoln and Guba [58] for evaluating qualitative research will be used in this study to increase credibility, transferability, dependability and confirmability. To ensure credibility in the study, reflective questioning will be used in both the qualitative interviews and focus groups. The research team will reflect on their interpretation of the participants′ answers, throughout the interviews/focus groups, to capture the meaning the participants were hoping to convey. Data (multiple key informants) and space (two recruitment sites) triangulation will also take place [59] and this will enhance credibility. Transferability of the results generated in the study will be achieved through ‘thick description’ [58]. This will entail providing explicit accounts of the experiences of the participants and not just detailing surface interpretations, but uncovering the meaning behind their feelings and actions as well [60]. Auditing will be used to establish dependability and confirmability of the study [58], such as the audit trail discussed within the analysis section.

### 2.5. Ethical Considerations

The study will be conducted in compliance with Good Clinical Practice Guidelines, with ethical and governance permissions from both recruitment sites. In addition, fundamental principles of good practice, including the provision of user-friendly information sheets, written informed consent, voluntary participation, the opportunity to withdraw from the study at any time and confidentiality and data protection procedures, will be applied as a minimum standard. As sensitive issues may be identified and discussed during the semi-structured interviews and focus groups, it is acknowledged that potential respondents may become distressed/upset. The research team will develop and use a distress protocol [61] within this study. As part of this distress protocol, post-interview, all patients and bereaved carers will be provided with information packs on support services. If required, they can self-refer to these services. Additionally, with participants’ consent, general practitioners will be advised that they have taken part within the study. As it is anticipated that interviews may be conducted in patients and carers homes (subject to COVID-19 restrictions), a lone worker policy [62] will be used within the study.

## 3. Discussion

This paper describes the study protocol of an evidenced informed service model of integrated palliative care for people living with an SMI. The main intention is that through raising awareness and facilitating the co-design of an evidenced based service model, the needs of service users and their caregivers from this marginalised overlooked group will be recognised and responded to within the model of integrated palliative care.

Globally mental health is a burden, in the United Kingdom, one in six of the population will require treatment for mental illness in their lifetime [63]. Large inequalities and gaps in care of people with mental illness exist, and this is a key indicator for health and wellbeing [15]. This research plans to significantly address health inequalities for people with SMI by illuminating the experiences of people with SMI, and bereaved caregivers and health care professionals to develop a co-designed service model of integrated palliative care. This will be done collaboratively with key stakeholders within the expert reference group, which includes people who experience these disparities in relation to accessing palliative care, bereaved caregivers and professional caregivers. It is anticipated that because of this study, service provision for people with SMI in need of palliative care will see a cultural shift in both public and professional awareness in relation to this vulnerable group.

## Figures and Tables

**Table 1:** Inclusion and exclusion criteria for Phase 3 participants.

**People with Severe Mental Illness.**	
**Inclusion Criteria**	**Exclusion Criteria**
Diagnosis of severe mental illness (as per operational definition)	No diagnosis of severe mental illness
Currently receiving palliative care	Not receiving palliative care
Over 18 years of age	Under 18 years of age
Capacity to take part in the study—as determined by the clinical gatekeeper	Does not have capacity to take part—as determined by the clinical gatekeeper
Can understand and speak English	Not fluent in English
**Caregivers**	
**Inclusion Criteria**	**Exclusion Criteria**
Bereaved caregiver of a patient with severe mental illness who has died 6–18 months earlier	Bereaved carer of patient who had died and not had a severe mental illness
Over 18 years of age	Under 18 years of age
Can understand and speak English	Not fluent in English

**Table 2:** Inclusion and exclusion criteria for Phase 4 participants.

Inclusion Criteria	Exclusion Criteria
Healthcare professionals involved in the care of people who have a severe mental illness and or palliative care	Healthcare professionals not involved in either severe mental illness or palliative care delivery
Employed within the recruiting Health and Social Trusts	Have a non-permanent contract with the Health and Social Trust, e.g., agency staff
Have an appropriate professional qualification in their field of work	Not fluent in English
Can understand and speak English	

## Data Availability

Data is not yet avialable in this study.

## References

[1] World Health Organisation Mental Disorders. http://www.who.int.

[2] Woods A., Willison K., Kington C., Gavin A. (2008). Palliative care for people with severe persistent mental illness: A review of the literature. Can. J. Psychiatry.

[3] Knippenberg I., Zaghouli N., Engels Y., Vissers K., Groot M. (2020). Severe mental illness and palliative care: Patient semistructured interviews. BMJ Support. Palliat. Care.

[4] Chochinov H.M., Martens P.J., Prior H.J., Kredentser M.S. (2012). Comparative health care use patterns of people with schizophrenia near the end of life: A population-based study in Manitoba, Canada. Schizophr. Res..

[5] Burton M.C., Warren M., Cha S.S., Stevens M., Blommer M., Kung S., Lapid M.I. (2014). Identifying Patients in the Acute Psychiatric Hospital Who May Benefit From a Palliative Care Approach. Am. J. Hosp. Palliat. Med..

[6] Wilson R., Hepgul N., Higginson I.J., Gao W. (2020). End-of-life care and place of death in adults with serious mental illness: A systematic review and narrative synthesis. Palliat. Med..

[7] McNamara B., Same A., Rosenwax L., Kelly B. (2018). Palliative care for people with schizophrenia: A quality study of an underserviced group in need. BMC Palliat. Care.

[8] Trachsel M., Hodel M.A., Irwin S.A., Hoff P., Biller-Andorno N., Riese F. (2019). Acceptability of palliative care approaches for patients with severe and persistent mental illness: A survey of psychiatrists in Switzerland. BMC Psychiatry.

[9] Brown S., Miranda K., Mitchell C., Inskip H. (2010). Twenty-five year mortality of a community cohort with schizophrenia. Br. J. Psychiatry.

[10] Kredenster M.S., Martens P.J., Chochinov H.M., Prior H.J. (2014). Cause and rate of death in people with schizophrenia across the lifespan: A population based study in Manitoba. J. Clin. Psychiatry.

[11] Irwin K.E., Henderson D.C., Knight H., Pirl W.F. (2014). Cancer care for individuals with schizophrenia. Cancer.

[12] Rocque G.B., Cleary J.F. (2013). Palliative care reduces morbidity and mortality in cancer. Nat. Rev. Clin. Oncol..

[13] Amano K., Morita T., Tatara R., Katayama H., Uno T., Takagi I. (2015). Association between early palliative care referrals, inpatient hospice utilization, and aggressiveness of care at the end of life. J. Palliat. Med..

[14] National Health Service Addressing Inequalities in End of Life Care. http://england.nhs.uk.

[15] Department of Health (2018). Service Framework for Mental Health and Wellbeing 2018–2021.

[16] Cai X., Cram P., Li Y. (2011). Origination of medical advance directives among nursing home residents with and without serious mental illness. Psychiatr. Serv..

[17] Ewert B., Hodiamont F., van Wijngaarden J., Payne S., Groot M., Hasselaar J., Menten J., Radbruch L. (2016). Building a taxonomy of integrated palliative care initiatives: Results from a focus group. BMJ Support. Palliat. Care.

[18] Brazil K. (2018). A Call for Integrated and Coordinated Palliative Care. J. Palliat. Med..

[19] Shefer G., Henderson C., Howard L.M., Murray J., Thornicroft G. (2014). Diagnostic overshadowing and other challenges involved in the diagnostic process of patients with mental illness who present in emergency departments with physical symptoms—A qualitative study. PLoS ONE.

[20] Donald E.E., Stajduhar K.I. (2019). A scoping review of palliative care for persons with severe persistent mental illness. Palliat. Support. Care.

[21] Sleeman K., de Brito M., Etkind S., Nkhoma K., Guo P., Higginson I.J., Gomes B., Harding R. (2019). The escalating global burden of serious-health related suffering: Projections to 2060 by world regions, age groups, and health conditions. Lancet Glob. Health..

[22] Ruggeri M., Leese M., Thornicroft G., Bisoffi G., Tansella M. (2000). Definition and prevalence of severe and persistent mental illness. Br. J. Psychiatry.

[23] Mulder C.L., van der Gaag M., Bruggeman R., Cahn W., Delespaul P.A.E., Dries P., Faber G. (2010). Routine Outcome Monitoring for patients with severe mental illness: A consensus document. Tijdschr. Voor Psychiatr..

[24] Delespaul P.H., de consensusgroep E.P.A. (2013). Consensus regarding the definition of persons with severe mental illness and the number of such persons in the Netherlands. Tijdschr. Voor Psychiatr..

[25] Creswell J.W. (2013). Research Design: Qualitative, Quantitative and Mixed Methods Approaches.

[26] Polit D.F., Beck C.T. (2016). Nursing Research: Principles and Methods.

[27] Capili B. (2020). How Does Research Start?. Am. J. Nurs..

[28] Bettany-Saltikov J., McSherry R. (2016). How to Do a Systematic Literature Review in Nursing: A Step by Step Guide.

[29] Addington Hall J., Mc Pierson C. (2001). After-death interviews with surrogates/bereaved family members: Some issues of validity. J. Pain Symptom Manag..

[30] McVeigh C., Reid J., Larkin P., Porter S., Hudson P. (2018). The provision of generalist and specialist palliative care for patients with non-malignant respiratory disease in the North and Republic of Ireland: A qualitative study. BMC Palliat. Care.

[31] Bakitas M., Ahles T.A., Skalla K., Brokaw F.C., Byock I., Hanscom B., Lyons K., Hegel M.T. (2008). Proxy perspectives regarding end-of-life care for persons with cancer. Cancer.

[32] Donnelly S., Prizeman G., Coimín D.Ó., Koran B., Haynes G. (2018). Voices that matter: End-of-life care on two acute hospitals from the perspective of bereaved relatives. BMC Palliat. Care.

[33] Trotter R. (2012). Qualitative research sample design and sample size: Resolving and unresolved issues and inferential imperatives. Prev. Med..

[34] Francis J.J., Johnston M., Robertson C., Glidewell L., Entwhistle L., Eccles M.P., Grimshaw J.M. (2010). What is an adequate sample size? Operationalising data saturation for theory-based interview studies. Psychol. Health.

[35] Sandelowski M., Given L.M. (2008). Theoretical Saturation. The SAGE Encyclopedia of Qualitative Research Methods.

[36] Walker S., Read S. (2011). Accessing vulnerable research populations: An experience with gatekeepers of ethical approval. Int. J. Palliat. Nurs..

[37] Green J., Thorogood N. (2009). Qualitative Methods for Health Research.

[38] Hudson P., Trauer T., Kelly B., O’Connor M., Thomas K., Summers M., Zordan R., White V. (2014). Reducing the psychological distress of family carers of home based palliative care patients: Longer term effects from a randomised controlled trial. Psycho-Oncol..

[39] Jamshed S. (2014). Qualitative research method-interviewing and observation. J. Basic Clin. Pharm..

[40] DeJonckheere M., Vaughn L.M. (2019). Semi structured interviewing in primary care research: A balance of relationship and rigour. Fam. Med. Community Health.

[41] Price B. (2002). Laddered questions and qualitative data research interviews. J. Adv. Nurs..

[42] Patton M.Q. (2002). Qualitative Research and Evaluation Methods.

[43] Morse J.M., Field P.A. (1996). Nursing research. The Application of Qualitative Approaches.

[44] Robinson J., Atkinson P., Delamont S., Cernat A., Sakshaug J.W., Williams R.A. (2019). Focus groups. SAGE Research Methods Foundations.

[45] Guest G., Namey E., McKenna K. (2016). How many focus groups are enough? Building an evidence base for nonprobability sample sizes. Field Methods.

[46] Tausch A.P., Menold N. (2016). Methodological Aspects of Focus Groups in Health Research: Results of Qualitative Interviews with Focus Group Moderators. Glob. Qual. Nurs. Res..

[47] Woodyatt C.R., Finnegan C.A., Stephenson R. (2016). In-person versus online focus group discussions: A comparative analysis of quality data. Qual. Health Res..

[48] McConnell T., Porter S. (2017). The experience of providing end of life care at a children’s hospice: A qualitative study. BMC Palliat. Care.

[49] Oliver S., Clarke-Jones L., Rees R., Milne R., Buchanan P., Gabbay J., Oakley A., Stein K. (2004). Involving consumers in research and development agenda setting for the NHS: Developing an evidenced-based approach. Health Technol. Assess..

[50] Donetto S., Tsianakas V., Robert G. (2014). Using Experienced-Based-Co-Design to Improve the Quality of Healthcare: Mapping Where We Are Now in Establishing Future Directions.

[51] University of Wisconsin-Extension Program Development and Evaluation: Logic Models. https://fyi.unexpected.edu/programdevelopment/logic-models/.

[52] Glasgow R.E., McKay G., Piette J., Reynolds K.D. (2001). The RE-AIM framework for evaluating interventions: What can it tell us about approaches to chronic illness management?. Patient Educ. Couns..

[53] Medical Research Council Developing and Evaluating Complex Interventions.

[54] Braun V., Clarke V. (2006). Using thematic analysis in psychology. Qual. Res..

[55] Roller M.R., Lavrakas P.J. (2015). Applied Qualitative Research Design: A Total Quality Framework Approach.

[56] Chen C., Draucker C.B., Carpenter J.S. (2018). What women say about their dysmenorrhea: A qualitative thematic analysis. BMC Women’s Health.

[57] Houghton C., Murphy K., Meehan B., Thomas J., Brooker D., Casey D. (2017). From screening to synthesis: Using nvivo to enhance transparency in qualitative evidence synthesis. J. Clin. Nurs..

[58] Lincoln Y.S., Guba E.G. (1985). Naturalistic Inquiry.

[59] Nobel H., Hale R. (2019). Triangulation in research, with examples. Evid. -Based Nurs..

[60] Korstjens I., Moser A. (2018). Series: Practical guidance to qualitative research. Part 4: Trustworthiness and publishing. Eur. J. Gen. Pr..

[61] Dempsey L., Dowling M., Larkin P., Murphy K. (2016). Sensitive interviewing in qualitative research. Res. Nurs. Health.

[62] Perkins E., Gambles M., Houten R., Harper S., Haycox A., O’Brien T., Richards S., Chen H., Nolan K., Ellershaw J. (2016). The care of dying people in nursing homes and intensive care units: A qualitative mixed-methods study. Health Serv. Deliv..

[63] Living with Schizophrenia. https://livingwithschizophrenia.org.

